# Supplementary material for: Homeostatic and tumourigenic activity of SOX2+ pituitary stem cells is controlled by the LATS/YAP/TAZ cascade
Source: eLife. 2019 Mar 26;8:e43996. doi: 10.7554/eLife.43996 (PMC6461440; doi:10.7554/eLife.43996)
Supplement: Supplementary file 1. — Embryonic: p=0.3471, Chi-square test (two tailed). Postnatal: p=0.0003 (***), Chi-square test (two tailed). [file elife-43996-supp1.docx]

Lodge et al.

**Supplementary File 1**

|  | **Embryonic** | | **Postnatal** | |
| --- | --- | --- | --- | --- |
| **Genotype** | **Observed** | **Expected** | **Observed** | **Expected** |
| *Hesx1^+/+^;Yap^fl/fl^;Taz^+/+^* | 4  (13.3%) | 3-4  (12.5%) | 14  (13.1%) | 13-14  (12.5%) |
| *Hesx1^+/+^;Yap^fl/fl^;Taz^+/-^* | 7  (23.3%) | 7-8  (25%) | 35  (32.7%) | 26-27  (25%) |
| *Hesx1^+/+^;Yap^fl/fl^;Taz^-/-^* | 2  (6.7%) | 3-4  (12.5%) | 4  (3.7%) | 13-14  (12.5%) |
| *Hesx1^Cre/+^;Yap^fl/fl^;Taz^+/+^* | 1  (3.3%) | 3-4  (12.5%) | 15  (14.0%) | 13-14  (12.5%) |
| *Hesx1^Cre/+^;Yap^fl/fl^;Taz^+/-^* | 12  (40%) | 7-8  (25%) | 37  (34.6%) | 26-27  (25%) |
| *Hesx1^Cre/+^;Yap^fl/fl^;Taz^-/-^* | 4  (13.3%) | 3-4  (12.5%) | 2  (1.9%) | 13-14  (12.5%) |
| **Total** | 30 | | 107 | |
