# Supplementary material for: Homeostatic and tumourigenic activity of SOX2+ pituitary stem cells is controlled by the LATS/YAP/TAZ cascade
Source: eLife. 2019 Mar 26;8:e43996. doi: 10.7554/eLife.43996 (PMC6461440; doi:10.7554/eLife.43996)
Supplement: Supplementary file 2. — Embryonic: p<0.0001 (****), Chi-square test (two tailed). Postnatal: p<0.0001 (****), Chi-square test (two tailed). [file elife-43996-supp2.docx]

Lodge et al.

**Supplementary File 2**

|  | **Embryonic** | | **Postnatal** | |
| --- | --- | --- | --- | --- |
| **Genotype** | **Observed** | **Expected** | **Observed** | **Expected** |
| *Hesx1^+/+^;Lats1^fl/+^;Lats2^+/+^* | 2  (1.6%) | 3-4  (2.75%) | 4  (2.1%) | 5-6  (2.75%) |
| *Hesx1^+/+^;Lats1^fl/+^;Lats2^fl/+^* | 4  (3.3%) | 15-16  (12.5%) | 9  (4.7%) | 23-24  (12.5%) |
| *Hesx1^+/+^;Lats1^fl/+^;Lats2^fl/fl^* | 19  (15.4%) | 11-12  (9.75%) | 32  (16.8%) | 18-19  (9.75%) |
| *Hesx1^+/+^;Lats1^fl/fl^;Lats2^+/+^* | 1  (0.8%) | 3-4  (2.75%) | 3  (1.6%) | 5-6  (2.75%) |
| *Hesx1^+/+^;Lats1^fl/fl^;Lats2^fl/+^* | 10  (8.1%) | 15-16  (12.5%) | 11  (5.8%) | 23-24  (12.5%) |
| *Hesx1^+/+^;Lats1^fl/fl^;Lats2^fl/fl^* | 14  (11.4%) | 11-12  (9.75%) | 44  (23.0%) | 18-19  (9.75%) |
| *Hesx1^Cre/+^;Lats1^fl/+^;Lats2^+/+^* | 9  (7.3%) | 3-4  (2.75%) | 17  (8.9%) | 5-6  (2.75%) |
| *Hesx1^Cre/+^;Lats1^fl/+^;Lats2^fl/+^* | 23  (18.7%) | 15-16  (12.5%) | 30  (15.7%) | 23-24  (12.5%) |
| *Hesx1^Cre/+^;Lats1^fl/+^;Lats2^fl/fl^* | 9  (7.3%) | 11-12  (9.75%) | 7  (3.7%) | 18-19  (9.75%) |
| *Hesx1^Cre/+^;Lats1^fl/fl^;Lats2^+/+^* | 9  (7.3%) | 3-4  (2.75%) | 10  (5.2%) | 5-6  (2.75%) |
| *Hesx1^Cre/+^;Lats1^fl/fl^;Lats2^fl/+^* | 18  (14.6%) | 15-16  (12.5%) | 24  (12.6%) | 23-24  (12.5%) |
| *Hesx1^Cre/+^;Lats1^fl/fl^;Lats2^fl/fl^* | 5  (4.1%) | 11-12  (9.75%) | 0  (0%) | 18-19  (9.75%) |
| **Total** | 123 | | 191 | |
